# Impact of Static Magnetic Field on the Antioxidant Defence System of Mice Fibroblasts

**DOI:** 10.1155/2018/5053608

**Published:** 2018-03-29

**Authors:** Marek Glinka, Stanisław Gawron, Aleksander Sieroń, Katarzyna Pawłowska-Góral, Grzegorz Cieślar, Karolina Sieroń

**Affiliations:** ^1^Research and Development Centre of Electrical Machines “Komel”, 188 Rozdzienskiego St., 40-203 Katowice, Poland; ^2^Department of Internal Medicine, Angiology and Physical Medicine in Bytom, School of Medicine with Division of Dentistry in Zabrze, Medical University of Silesia in Katowice, 15 Batorego St., 41-902 Bytom, Poland; ^3^Department of Food and Nutrition in Sosnowiec, Medical University of Silesia in Katowice, 8 Jednosci St., 41-200 Sosnowiec, Poland; ^4^Department of Physical Medicine, Chair of Physiotherapy, School of Health Sciences, Medical University of Silesia, Medykow 12 St., 40-752 Katowice, Poland

## Abstract

Results of research assessing the biological impact of static magnetic fields are controversial. So far, they have not provided a clear answer to their influence on cell functioning. Since the use of permanent magnets both in everyday life and in industry becomes more and more widespread, the investigations are continued in order to explain these controversies and to evaluate positive applications. The goal of current work was to assess the impact of static magnetic field of different intensities on redox homeostasis in cultures of fibroblasts. The use of permanent magnets allowed avoiding the thermal effects which are present in electromagnets. During the research we used 6 chambers, designed exclusively by us, with different values of field flux density (varying from 0.1 to 0.7 T). We have noted the decrease in the activity of superoxide dismutase (SOD) and glutathione peroxidase (GPx). The static magnetic fields did not modify the energy state of fibroblasts— adenosine triphosphate (ATP) concentration was stable, as well as the generation of malondialdehyde (MDA)—which is a marker of oxidative stress. Results of research suggest that static magnetic fields generated by permanent magnets do not cause oxidative stress in investigated fibroblasts and that they may show slight antioxidizing activity.

## 1. Introduction

During last decades the interest in impact of magnetic fields on life processes in living organisms and possible dangers induced by these fields has considerably increased. The issue of variable magnetic fields' influence on living organisms and their life functions was undertaken by many researchers, and the results differ depending on field intensity and frequency used in the tests. The steady development of electrical and electronic engineering processes and decreasing prices of permanent magnets (PM) have resulted in increased application of PMs in industry. This means that life organisms are exposed to magnetic fields for longer time intervals. At present, neodymium magnets (NdFeB) are used most often. These magnets make it possible to manufacture electrical machines characterized by best parameters (highest efficiency and power density) in relation to other types of machines. The result is that these machines are used more and more widely, in particular in car industry (electrical and hybrid vehicles), as well as in household appliances, hence the need for extensive research aimed at assessing magnetic fields' impact on different life factors in living organisms. Our previous experience with evaluation of variable magnetic fields' influence on life functions of fibroblasts and their antioxidizing activity has directed out interest towards permanent magnets [1, 2].

Initial research on impact of magnetic fields on fibroblasts was undertaken by Murray and Farndale. They assessed fibroblast proliferation in low frequency magnetic field and ability of fibroblasts to produce specific types of collagen. They did not find significant differences in proliferation of cells subjected to low frequency magnetic fields and type of produced collagen. However, they noted symptomatic increase in the quantity of produced collagen, which is probably due to influence of discussed fields on metabolism of cyclic AMP [3].

The effects of low frequency magnetic fields (*f* < 50 Hz) on mice fibroblasts and human promyelocytes proliferation were assessed by Schimmelpfeng and Dertinger [4]. The sham group in the research consisted of cells exposed to static magnetic field. No significant differences between the effects of static and variable magnetic fields were identified. However, the fields' parameters were not uniform, the selection was accidental, and cell cultures were not placed in homogeneous fields.

The majority of research on static magnetic fields' impact on free radical processes occurring in tissues provides contradictory data. Kula et al. [5] proved complete lack of any static magnetic field impact on superoxide dismutase, catalase, and glutathione peroxidase activity, as well as on malondialdehyde concentration in rat kidneys and livers. In turn in animals subjected to low frequency magnetic fields they detected increase in activity of antioxidizing enzymes cited above. Different results were obtained by Zmyślony et al. [6]. They showed that static magnetic fields increase peroxidation of lipid bilayers of cell membranes of microsomes in rat livers, so that they exert negative influence on different organs of living organisms (according to the researchers). It must, however, be noted, that their tests were conducted for low-intensity magnetic fields.

Zhao et al. [7] assessed concentration of cell ATP in human-hamster hybrid cells (A_L_), mitochondria-deficient (*ρ*^0^A_L_) cells, and double-strand break (DSB) repair-deficient (XRS-5) cells subjected to static magnetic field. They showed statistically significant differences occurring, depending on exposure time and field intensity. Very high field intensities used in the tests (8.5 T) caused significant decrease in ATP concentration and increase in the quantity of reactive oxygen species in the tested cells, while such differences were not observed in cultures subjected to lower field intensities (from 1 to 4 T).

In the research conducted by Shine et al. [8] the soya bean plant cells exposed to magnetic field of 200 mT showed significant increase in ROS production.

Nakahara et al. [9] did not prove any influence of static magnetic field (10 T) on growth and mitosis cycle in Chinese hamster ovary cells. They did not note any effect of strong SMF on cell growth, cycle distribution, or micronucleus frequency, but they observed an increase in the micronucleus formation induced by 4 Gy x-rays. The recently published research of Todorović et al. [10] showed that static magnetic fields of lower flux densities (50 mT) may significantly decrease activity of some antioxidizing enzymes in* Baculum extradentatum*. SOD and CAT activity decreased also in cultures subjected to variable magnetic fields, but GSH concentration in both groups did not change.

By analyzing previous research we tried to find the reason for such great diversity of various results. The investigations are hard to compare since there is no data provided as to the physical characteristics of static magnetic fields and types of magnets used; moreover, field intensities applied differed very widely.

The study presented here was conducted to determine whether the static magnetic fields generated by permanent magnets modify the growth of the fibroblasts' culture, activity of antioxidant enzymes, and intensity of lipid peroxidation. We decided to use magnets generating high but not extremely high values of field intensity.

The work was aimed at assessing the impact of continuous exposure of fibroblasts to static magnetic field, generated by permanent magnets. We made an evaluation of the redox balance in fibroblasts by

- determining activity of antioxidizing enzymes, such as superoxide dismutase (SOD) and glutathione peroxidase (GPx),
- determining activity of glutathione reductase (GR), the enzyme cooperating with GPx and largely responsible for proper cell concentration of reduced glutathione,
- determining the value of total antioxidant status (TAS),
- determining concentration of malondialdehyde (MDA), which is generally recognized as indicator of the intensity of free radical oxidation of fatty acids,
- determining ATP concentration, in order to assess metabolic state of fibroblasts.

## 2. Materials and Methods

### 2.1. Test Chamber for Static Magnetic Field Generation

The tests were run using a chamber designed and built by the authors. The detailed project and preliminary reports of the research were presented at 34th and 35th Bioelectromagnetics Society Annual Meeting in Halifax, Canada, and Brisbane, Australia, and subsequently published [11]. It has been granted protection under patent number P.396649 registered by Polish Patent Office.

**Figure 1:**
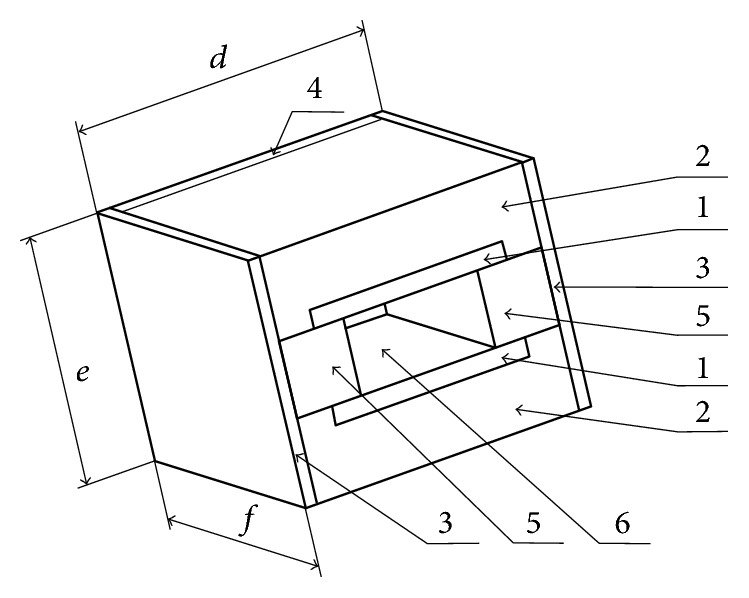
Cell culture chambers: chamber A, dimensions: *d* = 98 mm, *e* = 80 mm, *f* = 104 mm; chamber B, dimensions: *d* = 98 mm, *e* = 110 mm, *f* = 104 mm.

The details of construction are presented in Figure 1. In short, chamber** 6** consists of air-filled space, with artificial magnetic field present; this will hold culture flask** 9**. Chamber** 6** consists of ferromagnetic yoke** 2** and permanent magnets** 1**. The ferromagnetic yoke** 2** constitutes the bottom and cover of chamber** 6**, and permanent magnets** 1** are attached to it on the inside. Chamber** 6** is closed by side walls** 3**, back wall** 4**, and front wall** 7**. The inner dimensions of chamber** 6** are set by nonmagnetic distance plates** 5**, which are matched to culture flask dimensions** 9**: * a, b, c*. The front wall** 7 **of chamber** 6** is fitted with a window** 8**. Window** 8** dimensions * a, b* correspond to lateral dimensions * a*  and * b*  of culture flask** 9**, and dimension tolerance is +0.5 mm. Window is located centrally in chamber** 6**. At least some of walls** 3** or** 4** (or all of them) are ferromagnetic. Culture flask is of standard type; it is shaped like a rectangular bottle, made of transparent plastic, and closed with a plastic cap. Magnetic field intensity in chamber** 6** may be adjusted by exchanging permanent magnets** 1** for permanent magnets characterized by different parameters or size (thickness). If permanent magnets** 1** are thinner, then distance plates are placed between magnets** 1** and yoke** 2**. Their thickness must correspond to decrease in magnets thickness. Plates may be ferromagnetic or nonmagnetic. If plates are ferromagnetic, then change in magnetic field intensity in measurement zone is not significant, while with nonmagnetic plates it is greater. Sintered neodymium magnets were used to generate magnetic field (N42SH, CEIECSZ, Magtek, Shenzhen, China, with the following parameters: *B*_*r*_ = 1.27÷1.32 T, _B_*H*_C_ = 907 kA/m^3^, _J_*H*_C_ = 1592 kA/m^3^, (*BH*)_max_ = 318÷342 kJ/m^3^, maximum operating temperature 150°C).

**Figure 2:**
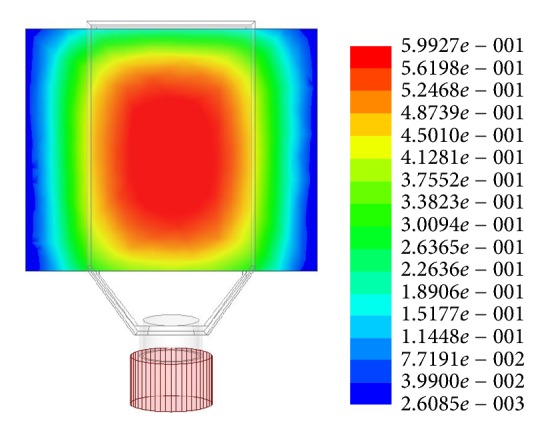
Magnetic flux distribution (flux density in T) in one of the test chambers, permanent magnets dimensions: 66 × 11 × 72 mm.

**Figure 3:**
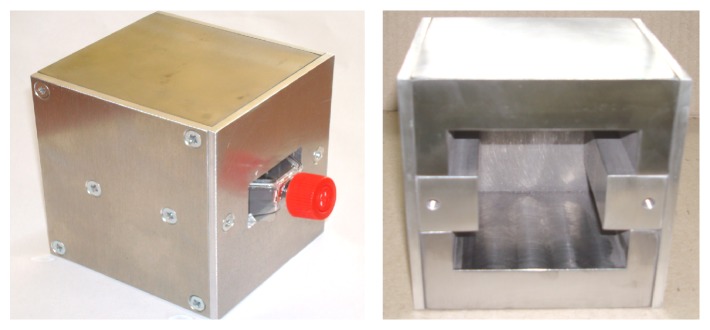
Photo of the test chamber.

The chamber was designed in such a way that the obtained magnetic field is homogenous all over the breeding surface. The magnetic flux density measurements were conducted with Magnetic Field Strength Meter Gauss-/Teslameter FH 55 and with Axial Hall Probe HS-AGB5-4805 (Magnet-Physik Dr. Steingroever GmbH, Cologne, Germany). The magnetic field characteristic across the culture surface for 11 mm thick magnet (0.6 T) is shown in Figure 2. The homogeneity of obtained field parameters for all of the culture surface must be noted.  Figure 3 shows the photo of one of the chambers.

Six types of chambers were used in the tests, with magnets of 4, 6, 8, 11, 15, and 20 mm thickness and flux densities 0.29; 0.40; 0.48; 0.58, 0.65, and 0.72 T, respectively. For “Placebo” chamber construction steel was used instead of magnet; the measurements conducted with gaussmeter showed overall flux density for this chamber equal to 0 T.

### 2.2. Fibroblast Culture Characteristics

Fibroblasts were isolated from tails and belly skin of 60-day-old mice, which were obtained from the Experimental Medicine Centre of Silesian Medical University in Katowice. This study was approved by the Local Animal Experimentation Ethics Committee. During experiment the animals were fed with standard feeding stuff and given tap water.

Fibroblasts were grown on Dulbecco MEM medium in cell culture flasks of 50 ml each and with breeding surface equal to 25 cm^2^. The flask was filled with 10 ml of medium enriched with inactive calf foetal serum so that final concentration was equal to 10%; antibiotics were also added: 1000 U of penicillin, 10 mg of streptomycin, and 25 *μ*g of amphotericin B per 1 ml medium. At the start of culture, each flask was filled with fibroblast suspension: 300 thousand per 1 ml of medium. After 24 hours from culture initiation the medium was completely replaced, and the culture was placed in static magnetic field. The incubations of test and control (sham) cultures were conducted in presence of air containing 5% (in terms of volume) of CO_2_, at temperature equal to 37°C, for 72 hours. Heraeus incubator was used. In order to obtain samples designed for fibroblast count in the cultures, the cells were subjected to trypsin. The cells dislocated from breeding surface were washed and spinned, and the obtained fibroblast sediment was suspended in 1 ml of PBS.

### 2.3. Preparation of Cell Homogenate

At the termination of each treatment, the cells were washed twice with ice-cold phosphate-buffered saline (PBS). The fibroblasts were mechanically homogenized using an Ultra-Turrax homogenizer (IKA Labortechnik, Staufen, Germany), in a flask placed on ice. The homogenization time was experimentally established, by assessing the effectiveness of the homogenization under a microscope. The resultant homogenates were then used in subsequent analyses.

All the studied biochemical parameters were recalculated for 10^6^ cells. All reagents were purchased from Sigma-Aldrich (St. Louis, MO, USA).

### 2.4. Superoxide Dismutase (SOD) Activity Assay

SOD activity was estimated according to the method of Beauchamp and Fridovich [12]. This method employs xanthine and xanthine oxidase to generate superoxide radicals which react with 2-(4-iodophenyl)-3-(4-nitrophenol)-5-phenyltetrazolium chloride (INT) to form a red formazon dye. The superoxide dismutase activity is then measured by the degree of inhibition of this reaction, and the absorbance at 340 nm was recorded for calculation of SOD activity. One unit (U) of SOD is that which causes a 50% inhibition of the rate of reduction of INT under the conditions of the assay. Activities of SOD were calculated from a standard curve and exposed as activity to 10^6^ fibroblasts.

### 2.5. Glutathione Peroxidase (GPx) Activity Assay

The GPx activity was measured by the method of Paglia and Valentine [13]. In this method glutathione peroxidase (GPx) catalyzes the oxidation of glutathione (GSH) by cumene hydroperoxide. In the presence of glutathione reductase and NADPH the oxidized glutathione (GSSG) is immediately converted to the reduced from with a concomitant oxidation of NADPH to NADP^+^. The decrease in absorbance at 340 nm was measured. Activities of GPx were recalculated to 10^6^ fibroblasts.

### 2.6. Glutathione Reductase (GR) Activity Assay

The GR activity was measured by the method of Goldberg and Spooner [14]. In this method glutathione reductase (GR) catalyzes the reduction of glutathione (GSSG) in the presence of NADPH, which is oxidized to NADP^+^. The decrease in absorbance at 340 nm was measured. Activities of GR were recalculated to 10^6^ fibroblasts.

### 2.7. Lipid Peroxidation Assay

Lipid peroxidation (as malondialdehyde, MDA) level was measured by the method of Gérard-Monnier et al. [15]. The method is based on the reaction of a chromogenic reagent, N-methyl-2-phenylindole (NPMI), with MDA at 45°C. One molecule of MDA reacts with 2 molecules of NPMI to yield a stable carbocyanine dye with a maximum absorption at 586 nm. In the MDA assay, a calibration curve is prepared using the MDA standard. The lipid peroxidation was expressed as MDA in nanomoles per 10^6^ fibroblasts.

### 2.8. Determination of Total Antioxidant Status (TAS)

The measurement of the total antioxidant status—a marker of nonenzymatic antioxidant system activity—was performed using the total antioxidant status test sets (Randox Laboratories Ltd., United Kingdom). The TAS method is based on reaction of ABTS (2,2′-azino-di-[3-ethylbenzthiazoline sulphonate]) with a peroxidase (metmyoglobin) and H_2_O_2_ to produce the radical cation ABTS®^•+^. This has a relatively stable blue-green color, which is measured at 600 nm. Antioxidants in the added sample cause suppression of this color production to a degree which is proportional to their concentration. Total antioxidant potential was exposed as micromoles per 10^6^ fibroblasts.

### 2.9. Adenosine Triphosphate (ATP) Assay

The ATP concentrations of test and control samples were determined with tests provided by Perkin-Elmer company. The determination procedure is based upon the reaction of ATP with D-luciferin catalyzed by luciferase and accompanied by emission of light.

The procedure was conducted directly in fibroblast cultures on 96-hole cell culture dishes, by measuring chemiluminescence with the help of multifunction plate reader Victor manufactured by Perkin-Elmer. ATP concentration for test cells was read from analytical (standard) curve constructed for ATP solution, for concentrations ranging from 1 to 10 *μ*mol/l, and then recalculated for 10^6^ fibroblasts.

### 2.10. Protein Assay

Protein content was determined using the method of Lowry et al. [16], with bovine serum albumin (BSA) as a standard.

### 2.11. Statistical Analysis

All data are expressed as the mean ± standard deviation of five separate experiments. An ANOVA and Tukey's post hoc test were used to evaluate the results of the experiments. The statistical calculations were performed using Statistica 10.0 (version 10.0, StatSoft, Cracow, Poland), and the statistical significance was defined at *p* < 0.05.

## 3. Results and Discussion

Activity of glutathione reductase did not change significantly (in the statistical sense) in the cultures subjected to SMF in relation to control cultures. Similarly, MDA and ATP concentrations as well as total antioxidant status (TAS) did not change in the investigated groups (Table 1).

**Figure 4:**
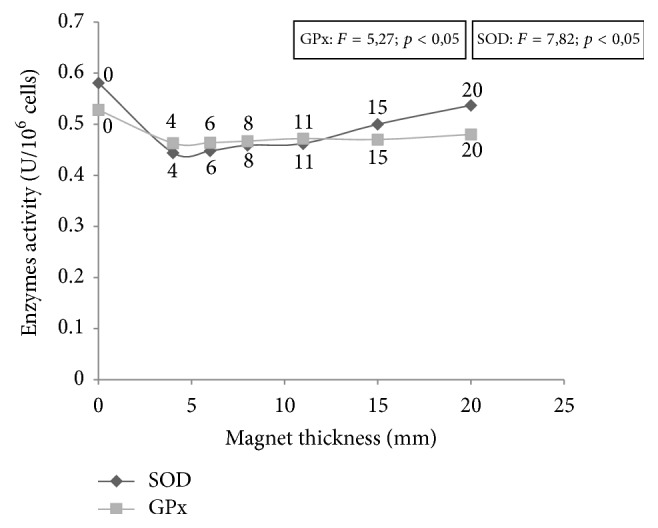
Effect of the static magnetic field on antioxidant enzymes (SOD and GPx) activity determined in fibroblasts cultures exposed to action of magnets with various thickness (4–20 mm) and control fibroblasts culture not exposed to magnet's action, with statistical evaluation (*F*, *p*—the values of ANOVA test).

However, we have obtained statistically significant decrease in activity of superoxide dismutase and glutathione peroxidase in all cultures subjected to static magnetic field action as compared to control cultures (Figure 4.) The chart shows greater variability in superoxide dismutase activity for different static field intensities, while the activity of glutathione peroxidase was more stable.

In mammals' antioxidizing endogenous system the superoxide dismutase (SOD) is quite common and plays a critical role. SOD is the principal enzyme protecting from the influence of superoxide anion-radical, which injures brain and other sensitive tissues [17]. It is generally accepted that the activity of superoxide dismutase increases temporarily in the tissues in response to oxidative stress. SOD activity in our research was changed in a statistically significant way in all cultures subjected to static magnetic field action in comparison to the sham exposed group (Figure 4). The greatest decrease in SOD activity was noted for chambers with lower static magnetic field intensities and the smallest for higher field intensities. In case of GPx such significant differences were not observed. Statistical analysis did not show any changes in malondialdehyde (MDA) concentration (Table 1). Keeping in mind MDA's role as an indicator of lipid peroxidation (in case of lipids—components of cell membranes), the lack of change of its concentration in the cultures proves the stability of fibroblast cell membranes. Similarly, ATP and TAS concentrations did not change in cultures subjected to static magnetic fields (as compared to the sham exposed groups), which suggests that SMF (within the range of intensity used in our research) do not influence the metabolic state of fibroblast culture [18].

The comparison of the impact of static and low frequency magnetic fields on fibroblast culture was conducted previously by Kula and Drózdz [19]. They investigated the influence of static and low frequency magnetic fields on growth in mice fibroblast culture, concentration of ^14^C-thymidine and protein, and the activity of antioxidant enzymes. They used electromagnet to produce static magnetic field and the cultures were exposed to it for several tens of minutes every day. They proved that there were no significant changes in the determined parameters in cell cultures subjected to static magnetic fields. However, in cultures exposed to low frequency magnetic fields fibroblast proliferation index increased, protein synthesis intensified, and DNA synthesis decreased. In turn research of Kula et al. [5] showed that static magnetic fields did not produce changes in catalase and superoxide dismutase activity, while variable magnetic fields inhibited this activity.

Since we used permanent magnets in our research, we were able to grow cell cultures under continuous exposure to static magnetic field. While culture homeostasis was kept constant, we avoided additional factors able to generate oxidative stress. Shine et al. during their research on impact of static magnetic field in soya bean cultures used electromagnets to generate static magnetic field and this required extra controlling of the thermal effects. Exposure time did not exceed one hour per day. They obtained significant increase of SOD activity in cultures, which according to the authors might be beneficial to the plant growth and their protection against free oxygen forms generated by external organisms [8]. Sahebjamei et al. [20] subjected calli of tobacco cell cultures to the impact of static magnetic field. Their investigations were conducted using water-cooled electromagnets generating magnetic field with intensity ranging from 0.5 *μ*T to 30 mT. On account of thermal effects produced by the device, the cultures were subjected to the static magnetic field action for 5 hours a day only. Increase in SOD activity and intensity of lipid peroxidation were observed and this testifies to the fact that static magnetic fields acting periodically upon plant cells may destabilize their cell membranes.

The developments in material engineering have made it possible to obtain static magnetic fields generated by permanent magnets and characterized by wide intensity range. This progress facilitates tests using homogenous fields and also helps to avoid thermal effects accompanying overheating of electromagnet circuits. We have attained such test conditions in our research, eliminating generation of additional oxidative stress for fibroblast culture which might be due to, for example, thermal effect.

Nowadays it is accepted that molecular action of static magnetic fields is caused by their acting upon nuclear spins of paramagnetic particles. Breaking of chemical bonds in case of components with paired electrons leads to emergence of unpaired forms and generation, for instance, of oxygen free radicals. The effect of static magnetic field may therefore be destructive, which is observed when culture exposure time is shorter [7, 8, 20], but it might not affect SOD activity at all, as seen in our own research. These differences in effects of constant low-intensity magnetic field impact on cell cultures were discussed by Martino [21]. He noted that even small changes in intensities of static magnetic fields may cause changes in different parameters of the cell culture (e.g., number of cells). He suggested that the observed effects might be explained on the basis of free radical mechanics. His research also refers to the earlier hypothesis stated by Steiner and Ulrich [22], who proposed that static magnetic fields may modulate transmission from triplet to single states, which might be of key importance in the kinetics of chemical reactions. Martino calls attention to the fact that differences in static magnetic field impact may depend on the effectiveness of eliminating the influence of geomagnetic field. The methods of culture and different devices (such as incubators) may strengthen or weaken this influence; total elimination may therefore lead to optimization of the obtained effects [21].

Design of our chamber and test procedures required measurements of static magnetic field value in the incubator. The measurements allowed us to evaluate the interaction of magnetic chambers for cell cultures, as well as mutual interference of fields generated by them. Details of chamber design and measurement results have been published before [11]. We did not observe any interference of static fields between the chambers; hence, it was possible to grow all cultures simultaneously in the incubator (within a given series of tests). The results of measurements of magnetic background in the incubator revealed values lower than those of geomagnetic field outside incubator. The size of the device and steel used for construction may attenuate geomagnetic field.

While the research lasted, we did not note statistically significant changes in fibroblast quantity in tested groups for all static magnetic field intensities. The previous research assessing the impact of static magnetic fields on growth rate of fibroblasts' cultures defines clearly the scope of SMF intensities used in the experiments. Sullivan et al. compared the influence of static low-intensity magnetic field on different types of fibroblasts' cultures. SMFs with intensities varying from 35 to 120 mT produced a significant halt in the cultures of foetal lung fibroblasts (WI 38 line) and adult skin fibroblasts; comparing both types of cells, this impact was greater in the group of foetal lung fibroblasts. Moderate values of SMF intensities (0.1 to 0.7 T) which were used in our research did not significantly influence growth rate in all cultures, as it was confirmed by statistical analysis [23]. Similar results were obtained by Wiskirchen et al. [24]. Their experiments were also conducted with foetal lung fibroblasts cultures, using permanent magnets with intensities 0.2, 1.0, and 1.5 T. In case of high-intensity fields (1.0 and 1.5 T) they did not observe any changes in the cultures during the experiment. Lack of changes in growth rate and cycle distribution of fibroblasts' cultures exposed to high-intensity SMF (up to 10 T) is confirmed by other reports [9]. It is suggested that low-intensity static magnetic field may impede the repair of DNA damage, and this is confirmed by research of Nüsse et al. [25]. These mechanisms are not yet completely recognized. The conclusions are drawn comparing the occurrence of whole chromosomes in cell micronuclei, after the cells have been subjected to high-intensity SMF (e.g., 10 T) with the occurrence of just single acentric fragments in fibroblast micronuclei, after the exposure of the cells to low-intensity SMF (below 0.1 T). The research on the impact of static magnetic fields on mice fibroblasts' culture in the fluoride ions environment was conducted by Kurzeja et al. [26]. Static magnetic fields (0.4 to 0.7 T) modified the energy state fibroblasts, causing an increase in the ATP concentration and a decrease in the MDA concentration. These results suggest that exposure to fluoride and static magnetic field improve the tolerance of cells to the oxidative stress caused by fluoride ions.

Practical implementation of results of the research shows further possibilities of investigating and application of static magnetic fields in medical diagnostic programs and, in particular, in nuclear magnetic imaging. Results of our and other researchers' work may constitute the basis for starting discussion about safety issues in magnetically levitating vehicles used for the public transport. Our research has proved that static magnetic fields do not cause changes in cell tolerance to oxidative stress; however, it is not possible to draw any conclusions as to their protective action against the harmful effects caused by oxygen free radicals. Number of experiments conducted is still not sufficient to assess the possibilities of SMF practical use or to draw conclusions about their safe application. Further research is necessary to evaluate biological aspects of static magnetic fields' impact, in particular of fields generated by permanent magnets.

## 4. Conclusions

Static magnetic fields can modify* in vitro* the redox homeostasis in mice fibroblasts; it does not cause oxidative stress in exposed fibroblast's cultures, but it stimulates slight antioxidant activity.

## Figures and Tables

**Table 1:** Effect of the static magnetic field on antioxidant defense parameters (ATP: adenosine triphosphate concentration, GR: glutathione reductase activity, MDA: malondialdehyde concentration, TAS: total antioxidant status) (mean values ± SD) in fibroblasts cultures exposed to action of magnets with various thicknesses (4–20 mm) and control fibroblasts culture not exposed to magnet's action, with statistical evaluation (*F*, *p*—the values of ANOVA test).

Magnet thickness	ATP [*μ*mol/10^6^ cells]	GR [U/10^6^ cells]	MDA [*μ*mol/10^6^ cells]	TAS [*μ*mol/10^6^ cells]
Control	7.5 ± 0.84	0.211 ± 0.028	0.335 ± 0.0073	0.323 ± 0.023
4 mm	6.65 ± 0.62	0.198 ± 0.004	0.338 ± 0.0012	0.268 ± 0.063
6 mm	6.82 ± 0.83	0.193 ± 0.024	0.321 ± 0.0075	0.280 ± 0.056
8 mm	6.56 ± 0.69	0.195 ± 0.026	0.319 ± 0.021	0.274 ± 0.058
11 mm	6.65 ± 0.89	0.194 ± 0.029	0.319 ± 0.023	0.271 ± 0.052
15 mm	7.09 ± 0.45	0.204 ± 0.046	0.306 ± 0.031	0.251 ± 0.048
20 mm	7.30 ± 0.52	0.208 ± 0.041	0.310 ± 0.040	0.253 ± 0.056
*F*	*0.941*	*1.846*	*0.517*	*0.935*
*p*	*0.48*	*0.172*	*0.667*	*0.487*

## Data Availability

The data used to support the findings of this study were provided by Research and Development Centre of Electrical Machines “Komel” in Katowice (Poland) under license and so cannot be made freely available. Access to these data will be considered by the author upon request, with permission of Research and Development Centre of Electrical Machines.
